# Online prediction of sustained muscle force from individual motor unit activities using adaptive surface EMG decomposition

**DOI:** 10.1186/s12984-024-01345-6

**Published:** 2024-04-04

**Authors:** Haowen Zhao, Yong Sun, Chengzhuang Wei, Yuanfei Xia, Ping Zhou, Xu Zhang

**Affiliations:** 1 School of Microelectronics, University of Science and Technology of China, Hefei, Anhui 230027 China; 2 Institute of Criminal Sciences, Hefei Public Security Bureau, Hefei, Anhui 230001 China; 3 Faculty of Biomedical and Rehabilitation Engineering, University of Health and Rehabilitation Sciences, Qingdao, Shandong 266024 China

**Keywords:** Muscle force prediction, Motor unit, Real-time EMG decomposition, Double-thread-parallel

## Abstract

Decoding movement intentions from motor unit (MU) activities to represent neural drive information plays a central role in establishing neural interfaces, but there remains a great challenge for obtaining precise MU activities during sustained muscle contractions. In this paper, we presented an online muscle force prediction method driven by individual MU activities that were decomposed from prolonged surface electromyogram (SEMG) signals in real time. In the training stage of the proposed method, a set of separation vectors was initialized for decomposing MU activities. After transferring each decomposed MU activity into a twitch force train according to its action potential waveform, a neural network was designed and trained for predicting muscle force. In the subsequent online stage, a practical double-thread-parallel algorithm was developed. One frontend thread predicted the muscle force in real time utilizing the trained network and the other backend thread simultaneously updated the separation vectors. To assess the performance of the proposed method, SEMG signals were recorded from the abductor pollicis brevis muscles of eight subjects and the contraction force was simultaneously collected. With the update procedure in the backend thread, the force prediction performance of the proposed method was significantly improved in terms of lower root mean square deviation (RMSD) of around 10% and higher fitness (R^2^) of around 0.90, outperforming two conventional methods. This study provides a promising technique for real-time myoelectric applications in movement control and health.

## Introduction

Surface electromyogram (SEMG) is a neuro-electrophysiological signal formed with muscle contractions. It can be viewed as the algebraic summation of motor unit action potential (MUAP) trains from different active motor units (MU) [1], which are the basic components of the peripheral neuromuscular system [2]. The SEMG signal has been widely used as a non-invasive interface. Precise muscle force prediction from the SEMG signal is one of the representative myoelectric control techniques to decode motor intentions, which is of great significance for sports biomechanics, robotic control, and rehabilitation [3–5].

Previous researches have reported stable and reproducible performance of muscle force estimation utilizing global features of the SEMG signal such as root mean square (RMS) [6–8]. These amplitude-associated features have attracted much interest with various applications in machine learning [9]- [10] or deep learning networks [11]- [12]. However, by using these global features, the direct myoelectric control systems can be severely influenced by motion artifacts, fatigue, background noise, and action potential variations, leading to performance degradation [13–15].

With the recent development of electronic and sensing technologies, the use of high-density SEMG (HD-SEMG) has been rapidly developed to facilitate implementation of SEMG decomposition in the past ten years [16]. This enables resolution of the composite EMG signal into its constituent MUAP waveforms and MU spike trains (MUSTs) in a non-invasive way [17–19]. In addition, the advent of online SEMG decomposition in a two-stage framework makes its application in daily life within the bounds of possibility [20–24].

The extracted MU activities, including MU firing patterns and waveform characteristic, contain neural information embedded in the electrical activity of skeletal muscle [25]. On this basis, the muscle force can be predicted from the extracted MU activities, which shows superiority over the global features [26]-[27]. Generally, the myoelectric control methods that used MU activities are usually termed as MU-driven methods [28]. Among these MU-driven methods, Zheng et al. established an MU firing rate-force model based on a linear regression algorithm [22]. Furthermore, Tang et al. [29] and Li et al. [30] utilized deep learning networks to mine spatial information of MUAP waveforms to distinguish different MUs for performance improvement.

Although these MU-driven methods have been proved effective with great potential, prolonged muscle contraction has always been an inevitable limitation [22, 31]. In the online SEMG method, the decomposition accuracies decrease over time due to many factors such as variations in muscle fiber length and conduction velocity [32]- [33]. Researches have reported that a lower decomposition accuracy can lead to larger force estimation errors [34]. Extensive computations are required to alleviate performance degradation during prolonged contraction [31], which conflicts with the online implementation of force prediction. Finding a way to establish an appropriate balance remains a great challenge.

To address the limitation, we proposed a novel online muscle force prediction method using a neural network. A series of separation vectors were calculated in the training stage using offline progressive FastICA peel-off (PFP) method for decomposing sEMG data into corresponding MU activities. The MU activities were transferred into twitch force trains and a long short-term memory (LSTM) neural network was utilized to predict the muscle force. During the online stage, an adaptive online SEMG decomposition method based on double-thread-parallel computation was employed to precisely trace MU discharges in the prolonged contractions so that force can be accurately estimated in real time. This study offers a useful tool for online myoelectric application with wide potentials in movement control and health.

## Methods

### Subjects

This study involved eight subjects (six males and two females, age: 26.00 ± 1.89 years) without any known muscular injuries or neuromuscular disorders participated. The experimental protocol was approved by the Ethics Review Committee of University of Science and Technology of China (Hefei, Anhui, China, under Application No. 2022-N(H)-163, February 2022). All subjects gave their informed and written consent prior to any procedure for the experiments.

**Fig. 1:**
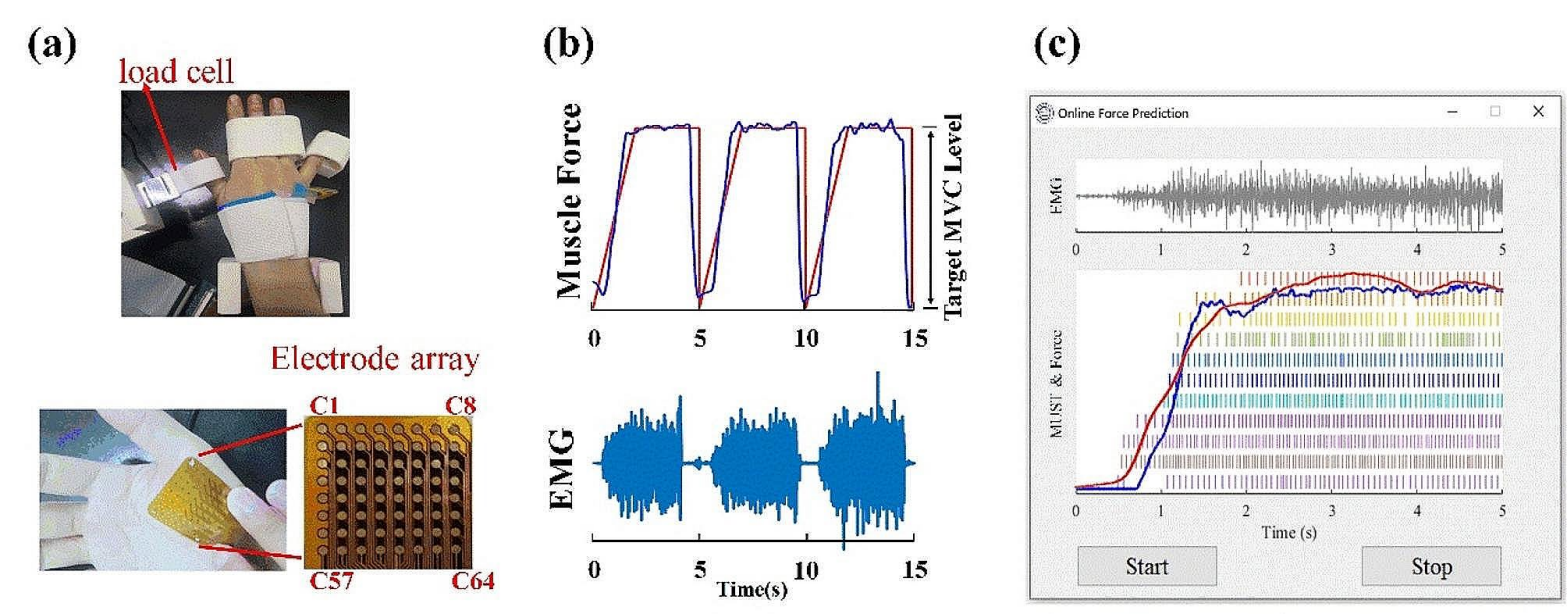
Experimental setup and protocol. (a). Apparatuses used for simultaneously recording thumb abduction force and HD-SEMG data. (b) Illustration of the force generation pattern with both the designed force curve (red line) and an actual recorded force curve (blue line) (c). The recorded EMG signals. (c) User interface of the software used for the online testing. There is an SEMG signal in one single channel as an example to exhibit the data stream on the top of the interface. Extracted MUSTs, measured force (red line) and predicted force (the blue line) are shown below

### Experimental protocols

The apparatus of the experiment is shown in Fig. 1a. A multi-channel electrode array arranged in 8 rows × 8 columns (FlexMatrix, Shanghai, China) was attached to the abductor pollicis brevis (APB) muscles on the dominant hand of a subject to record HD SEMG signals. Each electrode probe had a diameter of 2 mm with an inter-electrode distance of 4 mm between consecutive electrodes. At the beginning of the experiment, we tested all subjects’ maximum voluntary contraction (MVC) of the tested muscle (the maximum force of the thumb abduction contractions).

A series of contraction tasks were performed in the experiment, as shown in Fig. 1b. More specifically, the muscle force gradually increased from 0 to a targeted force level in 2s and then maintained at the targeted level for 3s in each trail of the contraction task so that a corresponding 5-s segment of SEMG signal could be collected. The targeted levels were set to 20% and 30% MVC. During the experiment, the subjects were required to sit comfortably. To prevent muscular interferences from the wrist or other fingers, a set of 3D-printed modules was fixed on appropriate positions of the table. The EMG recording system included a one-dimensional load cell (LDST-V-HY, Luckly Inc., Beijing, China) for recording EMG signals and muscle force simultaneously. The HD-SEMG signals in all channels were filtered through a 10-order Butterworth band-pass filter to reduce possible low-frequency or high-frequency interference. The bandwidth of the filter was 20–500 Hz. In addition, power line interference was removed through a 50 Hz second-order notch filter. All the recorded SEMG and force data were digitized via a 16-bit A/D converter (ADS1198, Texas Instruments, TX) at a sample rate of 2 kHz. The reliability of the data recording system has been proved in our previous studies [35]- [36].

#### Data collection for training

During this period, the subjects were instructed to perform the thumb abduction contraction task three times at 20% and 30% MVC, respectively. Sufficient rest was allowed for the subjects to avoid muscle fatigue. Data recorded in this experiment were used for calculating the MU separation vectors and training the muscle force model based on a deep learning network.

#### Online testing of the force prediction

To implement online testing, the data recording system was linked using a USB cable to a desktop computer with an Intel Core i9 CPU, 32 GB of RAM and an RTX3080Ti GPU for data transferring, recording, online SEMG decomposition and force estimation. Customized software was developed with the Python language using the deep learning framework termed Keras [37]. All of the procedures of the proposed method were implemented in the software. A graphical user interface (GUI) was also developed for interaction between the software and its users via the computer screen, as illustrated in Fig. 1 (c).

**Fig. 2:**
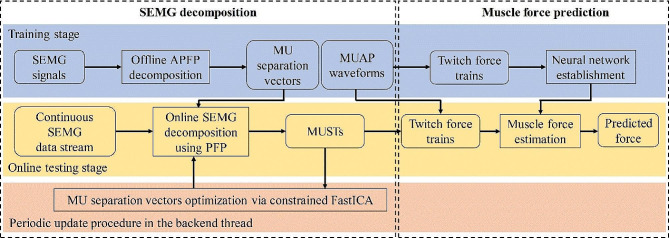
Block diagram of the proposed method for estimating muscle force using HD-SEMG data in real time. The framework of the proposed force prediction method contains the training stage (blue block) and the online testing stage (yellow block). The separation vectors were periodically update in the backend thread (red block) to maintain their validity

After the initialization, the subjects started online testing by repeating the force task mentioned above. For each of the subjects, 10 executions of the 5-s force task were required at 20% and 30% MVC. To meet the demand of online processing, the collected SEMG data stream was divided into a series of temporally overlapping windows with the window length and increment set at 1s and 0.2s, respectively. Real-time decomposition and force prediction were performed in a single 1-s window. During the online testing, the GUI showed a picture demonstration at a fixed interval of 0.2s, which was consistent with the increment of processing windows. The picture demonstration included the historical 5-s SEMG signals, extracted MUSTs, measured force and predicted force to guide task performance. After the test was completed, the overall accuracy was calculated and displayed on the screen. Figure 2 shows the framework of the proposed method.

### Training stage

The framework of the proposed method contained a training stage and online testing stage, which is consistent with the two-stage approach of online sEMG decomposition [21]. The pre-processing of the time-consuming computations and training can provide prior knowledge to benefit real-time applications. The training stage included the complete offline prework stage using the offline automatic PFP (APFP) [38] to obtain a series of separation vectors. The MUAP waveform of each MU was estimated following a least squares problem and all the waveforms were generated in 2D form.

Afterwards, the model for muscle force prediction needs to be established and trained. The establishment was two folds that are further explained in the following section.

#### Transfer of MUAP to twitch force

The twitch force model [39] can establish an electricity-to-force transformation process at MU level. In this model, the muscle force is described to be generated from the twitch forces of a series of activated MUs. Specifically, the twitch force of an activated MU is expressed as the second-order-system [39]:

1 $${f}_{i}\left(t\right)=\frac{{P}_{i}\bullet t}{{T}_{i}}\bullet {e}^{1-\frac{t}{{T}_{i}}}$$

where ${P}_{i}$ and ${T}_{i}$ represent the contraction time and the twitch force amplitude of the $i$th MU, respectively. In addition, an inverse power function is used to describe the relationship between ${P}_{i}$ and ${T}_{i}$:

2 $${T}_{i}={T}_{L}\bullet {\left(\frac{1}{{P}_{i}}\right) }^{\frac{1}{C}}$$

where ${T}_{L}$ is the maximal contraction time with a value set to 90ms and $c$ is a constant set to 4.2. ${P}_{i}$ is modeled to be linearly correlated with the peak-to-peak amplitude of the MUAP waveform. Generally, an activated MU usually discharges regularly. For the $j$th discharge time of the $i$th MU, the twitch force was described as:

3 $${f}_{i,j}\left(t\right)={g}_{i,j}\bullet \frac{{P}_{i}\bullet t}{{T}_{i}}\bullet {e}^{1-\frac{t}{{T}_{i}}}$$

where ${g}_{i,j}$ denotes the gain of the $j$th discharge time of the $i$th MU. It is defined as the ratio of the force to the firing rate. In this regard, the value of the gain was calculated as:

4 $${ISI}_{j}={t}_{i,j+1}-{t}_{i,j}$$

5 $${g}_{i,j}=\left\{\begin{array}{c}1, \frac{{T}_{i}}{{ISI}_{j}}\le 0.4\\ k\bullet \frac{1-{e}^{-2{\left(\frac{{T}_{i}}{{ISI}_{j}}\right)}^{3}}}{\frac{{T}_{i}}{{ISI}_{j}}}, otherwise\end{array}\right.$$

where $k$ is a constant and ${ISI}_{j}$ is the inter-spike interval (ISI), which means the time interval between two consecutive discharges of a single MU [40]. ${T}_{i}/{ISI}_{j}$ is the normalized stimulus rate.

The twitch force model was employed in each channel separately. To be consistent with the procedures of online decomposition, force prediction was performed on every 200-ms window (corresponding to a 0.2-s step of the 1-s window in the online decomposition, which contains 400 data points at a sampling rate of 2000 Hz). When there were N MUs decomposed in the online stage, the input SEMG feature map in a 400 × 8 × 8 × N data matrix was obtained as a basic sample for network training and testing. Each sample was labelled via the corresponding 100-point normalized force curve.

**Fig. 3:**
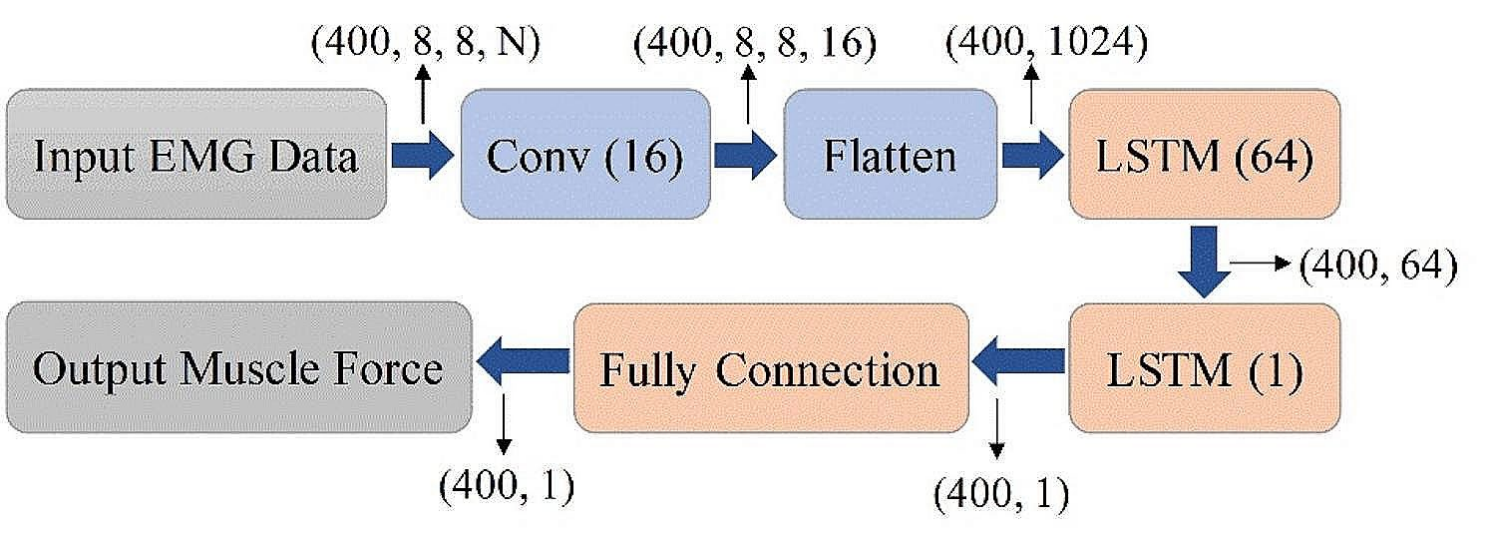
Architecture of the neural network used in this study. “conv” refers to a convolutional filter

#### Muscle force model based on neural network

Figure 3 shows the architecture of the network. One convolution layer was designed to take advantage of mining and characterize the spatial information from the 2D electrode array. The size of the convolutional filter was 3 × 3, the number of filters was 16, and the stride step was 1 × 1. The features obtained from the convolution layer were then processed using the typical LSTM to capture the long-term dependencies of data. The network contained two LSTM layers: one with 64 units and one with 1 unit. The final output of the predicted force (i.e. 400 × 1) was obtained from a fully connected layer. The whole network was trained with the Adam optimizer with a learning rate of 0.001. The root mean squared deviation (RMSD) was chosen as the loss function of the neural network and was calculated as:

6 $$RMSD=\sqrt{\frac{{\sum }_{i=1}^{n}{[\widehat{F}\left(t\right)-F(t\left)\right]}^{2}}{n}}\times 100\%$$

where $n$ denotes the number of the samples (i.e. 400). $\widehat{F}\left(t\right)$ and $F\left(t\right)$ represent the predicted force and the measured force, respectively.

To this end, the force prediction model was established. The initial 15-s SEMG signals were decomposed offline to obtain the MU activities and the twitch forces. These decomposition results were divided into a training set and a validation set at a ratio of 2:1. Then, the neural network was trained with the labels of measured force with a batch size of 32 and the network was trained for 150 epochs. This training stage is equivalent to the model training in the deep-learning approach.

### Online force prediction

**Fig. 4:**
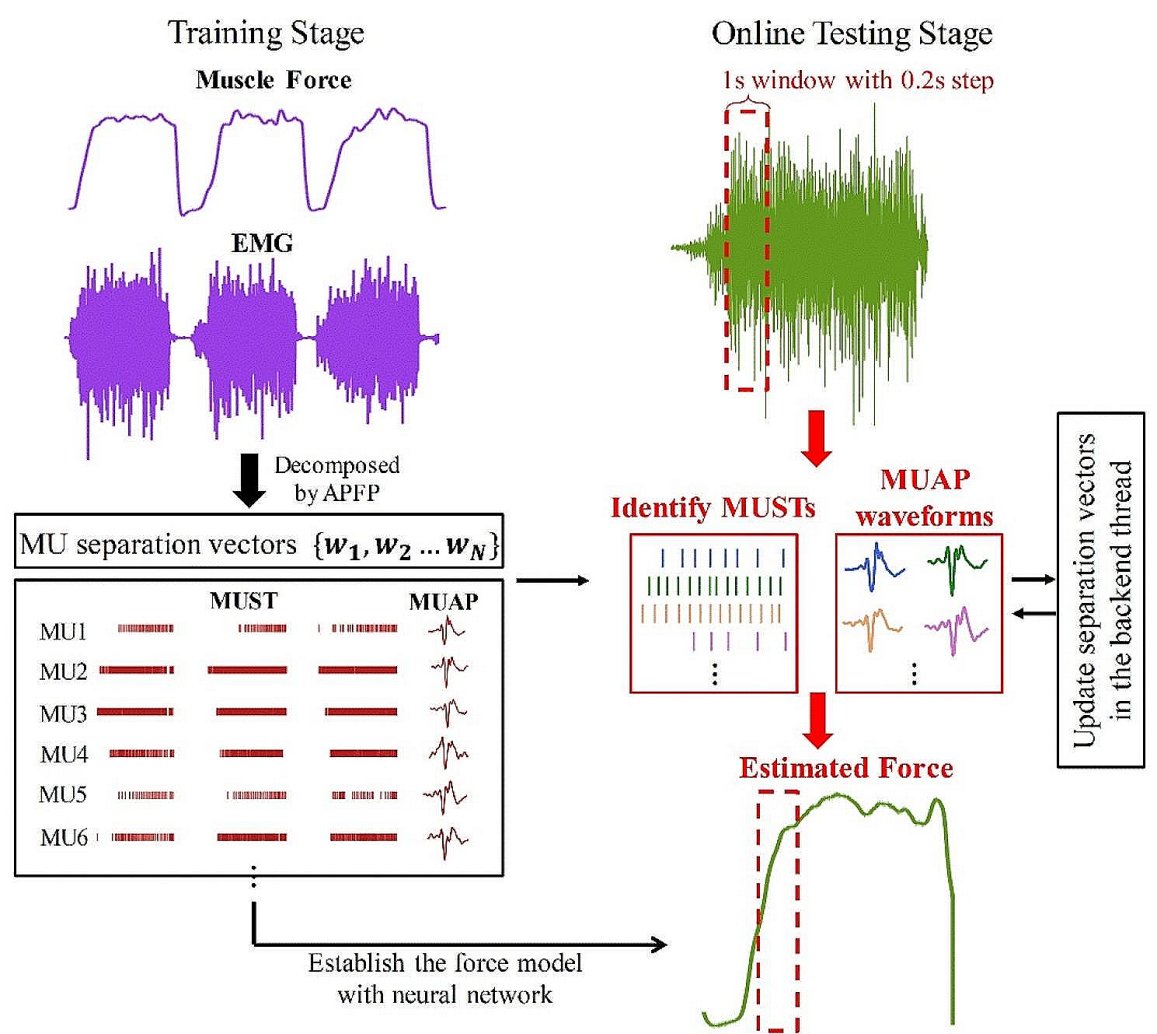
The illustration of the online force prediction process using the proposed method

During the online testing stage, the MUSTs were identified in short time windows from the extended and whitened SEMG data stream in real time, as shown in Fig. 4. The procedures of the MUST extraction kept consistent with our previous work using the successive multi-threshold Otsu algorithm [21]. In a single 1-s window, the MU firing events from the overlapping 0.8s were used to track the same MUs to ensure the continuity of decomposition results. The MU firing events from the last 0.2s were applied to estimate the force. More details of the processing and the corresponding parameters can be found in our previous study [21]. The online PFP method used in this study had the same settings reported in the same study [21].

After executing the online decomposition of SEMG signals, the MUSTs were continuously extracted and then transferred to multi-channel twitch force trains. These twitch force trains were input into trained neural network and the estimated muscle forces over windows were connected to form the resultant force.

To improve the performance of muscle force prediction, we designed a backend thread to update the MU separation vectors in the 5-second EMG segment at intervals of 10 s. This was done to adaptively update the MU separation vectors and maintain the validity of the vectors to precisely decode neural information. More specifically, the constrained FastICA was used to continuously track the same MUs with high precision in the backend thread. The spike trains of the identified MUs were used as constraints to drive the FastICA algorithm to converge rapidly (The detailed calculation steps of this constrained FastICA algorithm are given in previous studies [38, 41]). Therefore, for each MU, the separation vector can be effectively updated, and all the potential firing errors are believed to be corrected. After this update procedure, the original MU separation vectors were replaced by the updated version using the described procedure. The frontend thread applied the newly updated vectors to extract MUSTs and estimate muscle force in real time, processing in parallel with the backend thread.

### Performance evaluation

#### Performance metrics

The performance of force prediction was quantified by RMSD and the coefficient of determination (R^2^). For each of the different force levels, the RMSD and R^2^ values were averaged across subjects to represent the overall performance. In addition, we calculated the matching rates (MRs) of each individual MUST decomposed in the online decomposition stage with the ground-truth reference (the offline decomposition results). To better understand the effect of the backend update thread on the decomposition precision, the EMG data used in online testing stage were additionally decomposed by an offline APFP method [38]. The MR is defined as:

7 $$MR= \frac{2{N}_{com}}{{N}_{1}+ {N}_{2}}$$

where ${N}_{1}$ and ${N}_{2}$ are the number of spikes of the two spike trains decomposed online and offline, respectively, and ${N}_{com}$ is the number of common spikes. MR and the rate of agreement (RoA) are both commonly used criteria to assess the degree of matching of two spike trains and can effectively evaluate the decomposition performance [42]. Each MUST decomposed online was compared with each of the reference MUSTs, and the MUST achieving the maximum MR was selected from the reference to pair with the online decomposed MUST. This maximum MR was set as the decomposition accuracy for that MU.

To evaluate the real-time performance of the proposed method, we also calculated the time delay for processing the EMG data in a single 1-s time window, which was averaged all windows and all subjects.

#### Comparison methods

To demonstrate the outperformance of our method more comprehensively, two evaluations were performed. First, the RMSD and R^2^ values were evaluated with and without the backend update thread. In other words, the online force prediction without the update procedures could only use the MU information initialized in the training stage. This comparison method was denoted as the “no update” method, and the proposed method was termed as the “update” method.

Second, some conventional methods for muscle force prediction were also applied for comparison. One previously reported method using microscopic information was also adopted. This method employed the MU firing rate (FR) of the decomposed MUSTs to estimate force through a two-order polynomial regression model (termed as FR method) [22]. The FR in a single time window was calculated with a length of 250ms and increments of 50ms.

In addition, the RMS was also selected as a representative macroscopic feature for the conventional force prediction method based on EMG amplitude (termed as RMS method). The calculation of RMS was consistent with that of the FR, and the same two-order polynomial regression model was applied. Other settings of the comparison methods and the proposed method remained consistent or were fine-tuned for optimal performance.

### Statistical analysis

In order to evaluate the effect of the update procedure in the backend thread, two two-way ANOVAs were conducted on the RMSD percentage and R^2^, with both the update procedure (two levels: update and no update) and the method (three levels: the proposed method, FR method and RMS method) considered as the within-subject factors. The level of significant difference was set as *p* < 0.05. All statistical analyses were performed with SPSS software (ver. 22.0, SPSS Inc. Chicago, IL, USA).

## Results

### Time delay

The computational complexity of the proposed force prediction method was 0.106 ± 0.022s and was always less than 0.2s (the time length of the window increment). The processing time of the backend update thread was 4.56 ± 1.74s and was less than the 10s update period. The result demonstrated that the update strategy can meet the requirement for real-time processing [21, 22, 24]. Additionally, we also calculated the time delay of the comparison methods, which was much lower than the proposed method (FR method: 0.095 ± 0.014s, RMS method: 0.051 ± 0.018s).

**Fig. 5:**
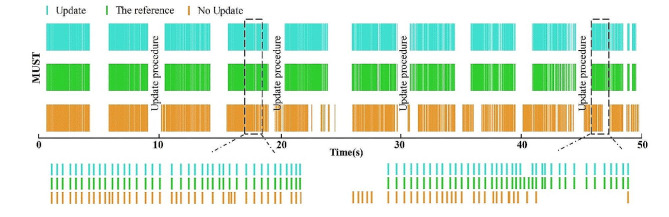
Comparison between the reference spike train from offline decomposition and the spike trains obtained through the online decomposition with and without update procedures. Two 2-second segments of spike trains are shown to illustrate more detailed spike timings

### Results of online identification of MUSTs

Figure 5 shows a comparison of the MUSTs of an identified MU obtained from the online decomposition process with and without update. The MU discharges derived from the decomposition process with update procedures matched well with the reference while there were many missing or erroneous discharges in the results of online decomposition without any update. Table 1 lists the online decomposition accuracies of all MUs across the subjects. For each subject, the accuracy was significantly improved by the update procedures.

**Table 1:** Decomposition accuracies (%) for experimental EMG across subjects

Subjects	S1	S2	S3	S4	S5	S6	S7	S8
Update	94.28 ± 4.51	95.91 ± 3.41	95.04 ± 4.28	93.52 ± 3.50	92.92 ± 6.71	91.92 ± 4.84	93.40 ± 4.87	93.18 ± 3.85
No update	87.24 ± 5.22	83.70 ± 3.41	82.93 ± 4.74	85.26 ± 4.88	84.80 ± 5.43	85.74 ± 3.74	86.17 ± 3.33	85.60 ± 5.35

**Fig. 6:**
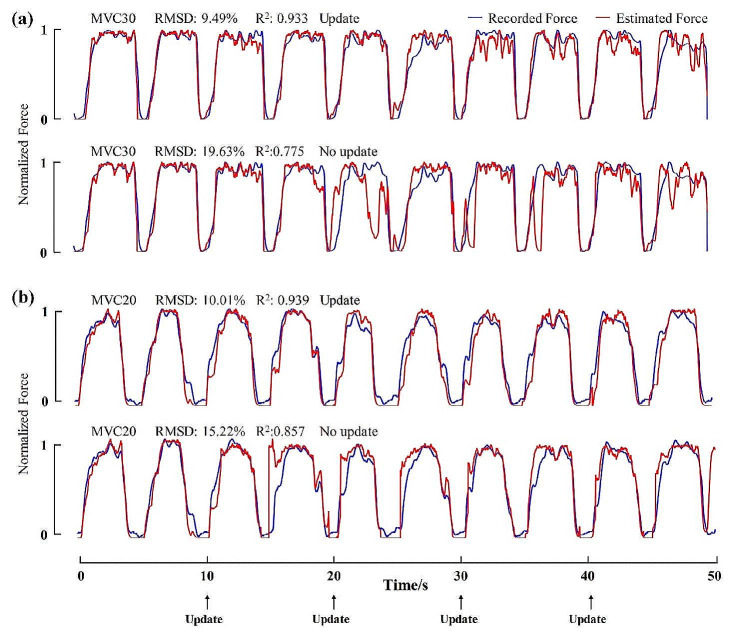
Force estimation results of a representative subject at 20% and 30% MVC using the proposed method with update and with no update, respectively

### Results of online force prediction

**Fig. 7:**
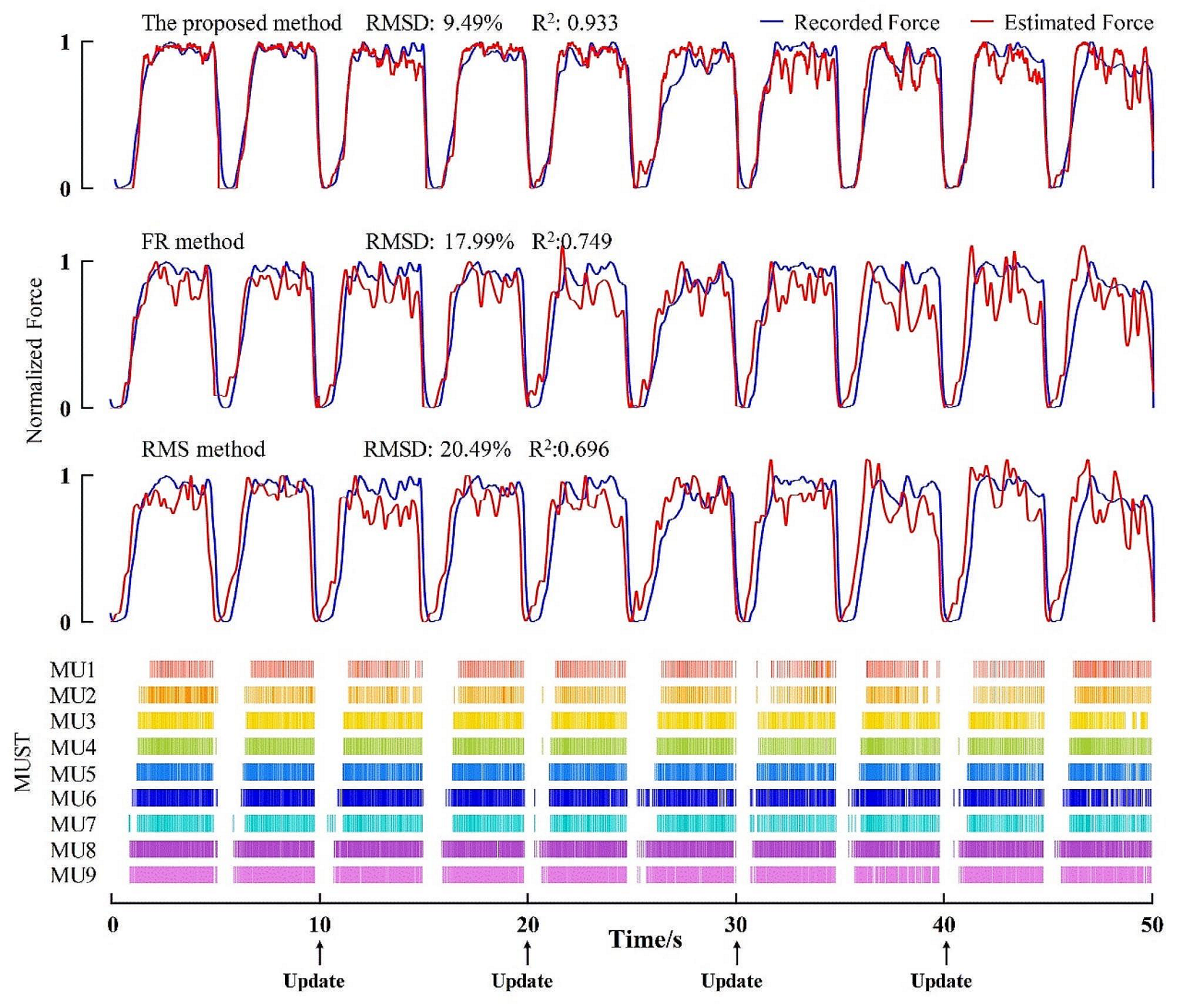
Force estimation results of a representative subject at 30% MVC using three common methods: the proposed method, the FR method and the RMS method

Fig. 6 shows examples of force estimation results of the proposed method with and without the update process at two contraction levels. The results show that the estimated force of the proposed method curve fitted the measured force curve much better than the “no update” method and the subtle force fluctuations can be effectively tracked with stable performance over time. Figure 7 exhibits representative results derived from using two comparison methods and the corresponding MUSTs. It can be observed that the estimated force curve of the proposed method had a better fit with lower RMSD and higher R^2^ than the other methods.

**Fig. 8:**
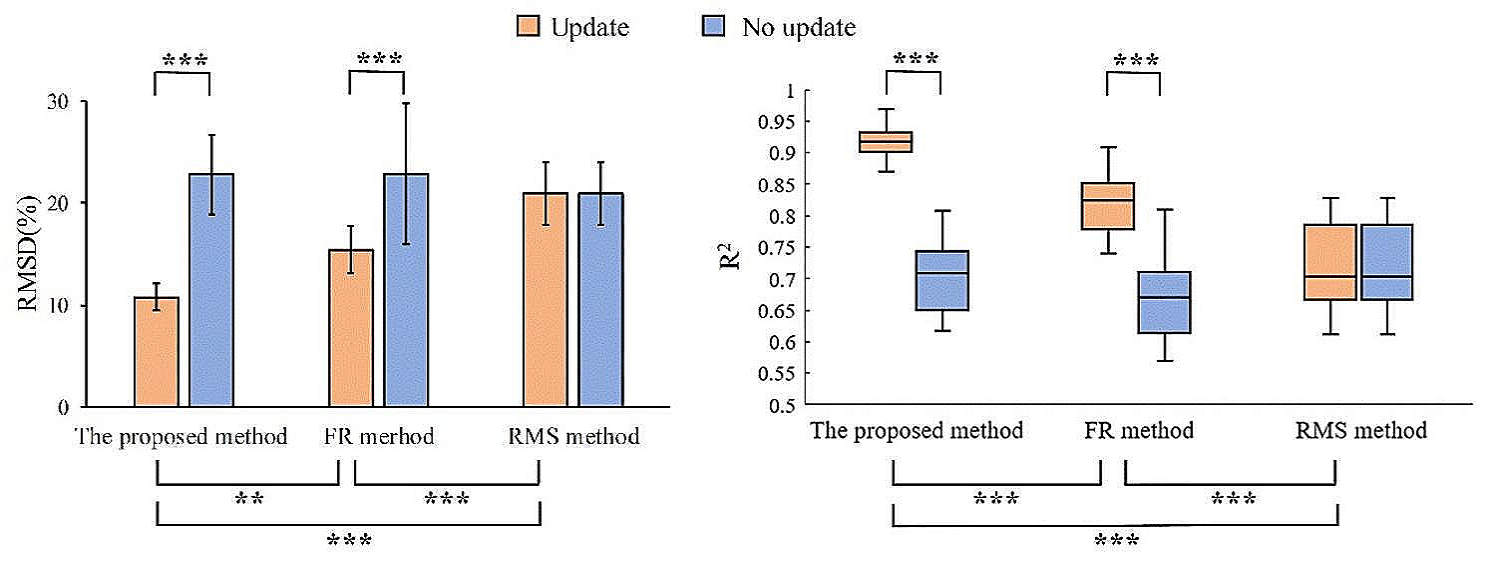
Figure 8. Force estimation performance evaluated by RMSD (left) and R^2^ (right) using the proposed method, the FR method and the RMS method. The effect of the update procedure is also shown with different colors (red and blue). * represents *p* < 0.05, ** represents *p* < 0.01, *** represents *p* < 0.001.

The performance of force estimation in terms of both RMSD and R^2^ metrics averaged over all subjects is shown in Fig. 8. The ANOVA showed that the update procedure significantly improved the RMSD of both the proposed method and the FR method (*p* < 0.001). Similarly, the ANOVA revealed that the R^2^ values of the proposed method and the FR method with the update procedure has a higher correlation with the measured force than those with no update (*p* < 0.001). The RMSD and R^2^ of the RMS method stayed the same with and without the update procedure. In addition, we also evaluated the force prediction performance of the proposed method and the two comparison methods. The results demonstrated a significantly smaller estimation error of the proposed method than the FR method (*p* < 0.05) and the RMS method (*p* < 0.001) when there was an update procedure. The proposed method also showed a higher R^2^ value of force prediction than the FR method (*p* < 0.001) and RMS method (*p* < 0.001).

## Discussion

This study presents a novel method for predicting muscle force from SEMG signals in real time. In the proposed method, the MU separation vectors were first calculated in the training stage and the corresponding MUAP waveforms were transferred into the twitch force trains. Meanwhile, the muscle force model that contained a twitch force model and a neural network was established and trained. During the online testing stage, one frontend thread obtained MUSTs from SEMG streams in real time and fed them into the trained network to estimate the corresponding muscle force. The other backend thread simultaneously refined the MU separation vectors at fixed intervals to provide precise MUSTs for the force model. Our results proved the feasibility and effectiveness of the proposed method, which can help develop a more useful neural interface technique decoding neural drive information from individual MU activities.

### Benefits of adaptive online decomposition

With the update procedure in the backend thread, high decomposition accuracy was maintained during prolonged muscle contractions, as shown in Table I. Moreover, significant improvement of force prediction was shown in our results, illustrating the effectiveness of the double-thread-parallel algorithm. This is due to the fact that long-term muscle contraction can lead to a negative effect on the muscle force prediction based on SEMG signals, which has been discussed in a previous study [22]. The prediction performance degradation can be explained by the decrease of the decomposition accuracy during prolonged contractions [31]. Due to the fact that MU discharges determine the time of the corresponding twitch force, it is essential to precisely extract MUSTs in the MU-driven force prediction method. If the errors of the MUST extraction accumulate over time, it can strongly compromise the force estimation. This limitation has not been effectively overcome in previous studies [22]. In this study, we combined a double-thread-parallel algorithm in the proposed method. Through the update procedure in the backend thread, the validity of separation vectors was maintained using the constrained FastICA algorithm, thus benefiting the process of force estimation. It is worth mentioning that the FR method could also benefit from the update procedures, because it is also highly dependent on the precise MU firing information. The results demonstrated the potential of our method to improve the performance of neural-machine interface systems that use MU discharge information. Furthermore, the time delay of our method kept in an acceptable range, and the complexity met the requirements for real-time performance, which also benefited from the double-thread parallel computation strategy.

In our previous study, a module was made for cross-trial MU identification and tracking in the offline framework of force estimation due to inconsistency of the offline SEMG decomposition across different trails [29]. Such inconsistency means that it cannot satisfy the prerequisites for introducing advanced deep neural networks in processing MU activities, so that it is necessary to sort the MUs into fixed categories with an MU tracking module [29]. In comparison, this module for MU clustering was not included in our proposed method. This can be explained that the MU separation vector used in the online stage has the capability of continuously tracking the same MU, which is the basic assumption of the two-stage approach for online SEMG decomposition [20–24]. In other words, the application of the two-stage approach into muscle force prediction can effectively overcome the cross-trial inconsistency of the offline MU-driven method. This is critical because the precise tracing of the same MU during long-term recordings significantly affects the force prediction performance and the update procedure in the proposed method helps maintain the validity of the separation vectors to make the proposed method more suitable for prolonged muscle contractions. In addition, the results fully demonstrated that the transfer of MU separation vectors enables the generalization of knowledge from the offline stage to the online stage using a deep neural network. Our study illustrated the unique advantage and significance of online SEMG decomposition applied into myoelectric control.

### Comparison with conventional methods

Compared with the microscopic neural drive information, global features such as the EMG amplitude are more accessible and it is straightforward to use these features for estimating muscle force. In our results, the neural-drive methods yielded the lowest RMSD and highest R^2^ and its performance was better than that of RMS-based method, which is consistent with the literatures [28–30]. Essentially, the correlation between SEMG signals and muscle force can be seemed as a ‘black box’ consisting of the activities of activated MUs, and the amplitude-based method is just an oversimplified description of the ‘black box’ [43]. In contrast, the neural drive information derived from the MU activities makes the force estimation process more analytical and transparent, by following the physiology of muscle movement. In addition, the results demonstrated the superiority of the proposed method over a common neural-drive method based on MU discharges, which has been extensively investigated both offline [34]-[36] and online [22, 28]. Although the processing time cost of the proposed method caused by the online SEMG decomposition was higher than the comparison methods, it significantly improved the performance of force prediction, which makes it a promising and worthwhile trade-off. The outperformance of our method can be explained by the reason that the different contributions of MUs were effectively distinguished by the means of mining their MUAP spatial information using deep learning in our proposed method. In contrast, the FR method only uses the firing rate of the composite MUSTs without any waveform information. Through periodical updates in the backend thread, the precise MU firing information can be provided to fully exploit the advantages of distinguishing MU, outperforming the other online MU-driven applications that only investigated the MU discharge information with a simplified machine learning algorithm [22, 28].

### Limitations

There were some limitations in the current study that need to be clarified. First, with the consideration of the update period, the peel-off procedure in the offline APFP method did not transfer into the online version due to the long processing time. The peel-off procedure can make full use of already identified MUs and help FastICA to find more MU source signals, which could provide a more comprehensive description of the activated MUs. Second, the SEMG signals were collected from only isometric contractions of APB muscles. More complex experimental paradigms including different muscles (such as the biceps brachii) and non-isometric conditions need to be validated. Third, the robustness of the method against electrode shift or other interferences needs to be improved for the practical neural interfaces. This problem also remains in research on online SEMG decomposition. Further research will be devoted to addressing these limitations.

## Conclusion

A novel method for predicting muscle force in real-time based on neural drive information from individual motor unit activities was presented in this paper. The MU separation vectors were obtained in the training stage and the waveforms of the decomposed MUs were transferred into twitch force trains. A neural network was established and trained to predict muscle force. During the online testing stage, the SEMG signals were decomposed in real time and the extracted MUSTs were utilized in the trained model to estimate muscle force. Moreover, a double-thread-parallel algorithm was integrated to periodically update the MU separation vectors to alleviate the performance degradation during prolonged contractions. Full validation was performed with experimental SEMG data, and the proposed method significantly improved the force estimation precision and outperformed the two common methods. This study offers a promising tool for predicting muscle force in real time, with a variety of applications in the neural interface techniques based on SEMG signals.

## Data Availability

No datasets were generated or analysed during the current study.

## Abbreviations

***MU***: motor unit
***SEMG***: surface electromyogram
***RMSD***: root mean square deviation
***R***^***2***^: fitness
***MUAP***: motor unit action potential
***RMS***: root mean square
***HD-SEMG***: high-density SEMG
***MUST***: MU spike train
***APB***: abductor pollicis brevis
***MVC***: maximum voluntary contraction
***GUI***: graphic user interface
***LSTM***: long short-term memory
***PFP***: progressive FastICA peel-off
***APFP***: automatic progressive FastICA peel-off
***MR***: matching rates
***RoA***: rate of agreement
***FR***: firing rate
